# Children's eating behavior: comparison between normal and overweight children from a school in Pelotas, Rio Grande do Sul, Brazil

**DOI:** 10.1016/j.rpped.2014.11.007

**Published:** 2015-03

**Authors:** Darlise Rodrigues dos Passos, Denise Petrucci Gigante, Francine Villela Maciel, Alicia Matijasevich

**Affiliations:** a Universidade Federal de Pelotas, Pelotas, RS, Brazil; b Universidade de São Paulo, São Paulo, SP, Brazil

**Keywords:** Obesity, Nutritional assessment, Feeding behavior, Child

## Abstract

**OBJECTIVE:**

To evaluate differences in children's eating behavior in relation to their nutritional status, gender and age.

**METHODS:**

Male and female children aged six to ten years were included. They were recruited from a private school in the city of Pelotas, Rio Grande do Sul, southern Brazil, in 2012. Children´s Eating Behaviour Questionnaire (CEBQ) subscales were used to assess eating behaviors: Food Responsiveness (FR), Enjoyment of Food (EF), Desire to Drink (DD), Emotional Overeating (EOE), Emotional Undereating (EUE), Satiety Responsiveness (SR), Food Fussiness (FF) and Slowness in Eating (SE). Age-adjusted body mass index (BMI) z-scores were calculated according to the WHO recommendations to assess nutritional status.

**RESULTS:**

The study sample comprised 335 children aged 87.9±10.4 months and 49.3% had normal weight (n=163), 26% were overweight (n=86), 15% were obese (n=50) and 9.7% were severely obese (n=32). Children with excess weight showed higher scores at the CEBQ subscales associated with "food approach" (FR, EF, DD, EOE, *p*<0.001) and lower scores on two "food avoidance" subscales (SR and SE, *p*<0.001 and *p*=0.003, respectively) compared to normal weight children. Differences in the eating behavior related to gender and age were not found.

**CONCLUSIONS:**

"Food approach" subscales were positively associated to excess weight in children, but no associations with gender and age were found.

## Introduction

The study of eating behavior plays a central role in the prevention and treatment of chronic diseases associated with poor nutrition.^1^ Among them is obesity, a main nutritional problem that constitutes a challenge in both developed and developing countries.^2^ In 2008, approximately 33% of Brazilian children between five and nine years old were overweight, of which about 14% were already obese.^3^

Studies have shown differences in several dimensions of eating behavior among children with and without excess weight.^4^^-^ 17 It is believed that overweight children are more responsive to external stimuli in the environment (e.g., flavor and color of food), demonstrate greater pleasure in eating and have lower responsiveness to satiety when compared to children with healthy weight, which causes them to eat larger amounts, and in the absence of hunger, thus demonstrating a greater interest in food.^4^^,^ 5 ^,^ 11 ^,^ 13 ^,^ 16 Moreover, they have the habit of eating in order to deal with different emotional states (happiness, anxiety and stress),^4^^,^ 5 ^,^ 13 they often drink sugary beverages during the day and eat more quickly.^14^^,^ 15 On the other hand, underweight children seem to be more selective in relation to food, consuming small meals, with a limited number of foods and more slowly, thus reflecting a lack of interest in food.^4^^,^ 13

It is known that eating behaviors are formed in the first years of life,^18^ and eating habits in adulthood are related to those learned in childhood.^19^ Additionally, changes in behavior with advancing age tend to be more difficult to be achieved.^20^ These situations demonstrate the importance of investigating eating behaviors at early ages and suggest that actions aimed at promoting healthy eating habits should focus with greater emphasis on children. Given the above, the aim of this study was to evaluate differences in eating behavior according to nutritional status, gender and age of children aged 6 to 10 at a private school in the city of Pelotas.

## Method

This is a cross-sectional study in a private school in the city of Pelotas, Rio Grande do Sul, carried out from May to June 2012. All children included in the study were 6 to 10 years old, enrolled in the 1^st^, 2^nd^ or 3^rd^ year of elementary school, whose parents or guardians gave their consent to participate in the study (total of 359 children). Children without anthropometric data were excluded from the sample.

The assessed outcome was eating behavior, assessed through the subjective perception of parents about their children's behavior by answering the CEBQ questionnaire (C*hildren's Eating Behaviour Questionnaire*),^4^ translated and validated for a sample of Portuguese children.^5^ This questionnaire contains 35 questions divided into eight subscales, so that four subscales investigate behaviors that reflect "interest in food" - Food Response (FR), Enjoyment of Food (EF), Desire to Drink (DD) and Emotional Overeating (EOE) - and the other four subscales reflect behaviors related to "lack of interest in food" - Emotional Undereating (EUE), Satiety Responsiveness (SR), Slowness in Eating (SE) and Food Fussiness (FF). Examples of questions contained in the questionnaire are: "Given the opportunity, my child would spend most of the time eating" (FR); "My child loves to eat" (EF); "Given the opportunity, my child would spend the day continuously drinking (soft drinks or sweetened juices)" (DD); "My child eats more when he/she is anxious" (EOE); "My child eats less when he/she is tired" (EUE); "My child feels full before finishing the meal" (SR); "My child eats more and more slowly throughout the meal" (SE), and "When given new foods my child initially refuses them" (FF).The questionnaires were sent by the teachers for parents or guardians to fill them out, and the answers were given using a Likert scale of five points, according to the frequency in which their children presented each behavior, with the score ranging from 1 to 5: never (1), rarely (2), sometimes (3), often (4) and always (5). The scores of questions that belonged to the same subscale were added up, so that each subscale had a mean value and standard deviation. In cases of unanswered questions, telephone contact was made to obtain the information.

At school, anthropometric measurements of weight and height were collected by previously trained and standardized nutritionists or nutrition students. Children were weighed with light clothes and barefoot, on a digital bioimpedance scale (Tanita Corporation of America, Inc., IL, USA) with a capacity of 150 kg and precision of 100 g. Height was measured with a portable vertical stadiometer (Alturexata^(r)^, MG, Brazil) with 213 cm in length and precision of 0.1 cm. The nutritional status of children was assessed using the z-score of body mass index for age (BMI/A) and classified according to the cutoffs proposed by the World Health Organization (WHO)^21^ into five categories: underweight, normal weight, overweight, obesity and severe obesity. The WHO Anthro Plus software (WHO Anthro Plus^(r)^, World Health Organization, GE, Switzerland) was used to calculate the z-score.^21^

The age variable was collected continuously in months and subsequently categorized into the following age groups: <7 years, between 7 to 7.9 years and 8 years or older. To evaluate parental educational level, we determined the percentage of parents who had finished college/university. 

The collected data were entered in duplicate in EpiData (EpiData^(r)^ Software and Templates, World Health Organization, GE, Switzerland), and all analyses were performed using Stata^(r)^ software, version 12.0. The descriptive analysis of the data was performed using mean and standard deviation for continuous variables, and proportions for categorical variables. Analysis of variance (ANOVA) was used to compare the mean score obtained in each of the CEBQ subscales according to the categories of different exposure variables (nutritional status, gender and age). Linear trend test was performed to demonstrate the variation in the scores of the CEBQ subscales in different categories of BMI z-score. Linear regression was performed to assess the association between BMI z-score/age and subscale means, controlling for potential confounders: gender and age of children and parents' level of schooling. The BMI z-score (categorized) was correlated to the variable gender with contingency tables. The respective prevalence of children was calculated for each category of BMI z-score and then compared between males and females. Statistical tests were based on the chi-square test. The significance level was set at 5%.

The project was approved by the Research Ethics Committee of the Faculdade de Medicina of the Universidade Federal de Pelotas, protocol number 26/2012. The free and informed consent form was signed by the children's parents or guardians.

## Results

A total of 335 children participated in the study, representing 93.3% of the students enrolled in the grades included in the study (total of denials: 6.7%). It was observed that 51.3% of the children were females, 94.3% were Caucasians, and most parents had a college/university level of schooling (75.7% of mothers and 63.7% of fathers of the total number of parents/guardians that completed the questionnaire). The mean age of the study population was 87.9 months (±10.4 months), and the three age groups were approximately the same size: <7 years (n=134), 7 to 7.9 years (n=119) and ≥8 years (n=82).

**Figure 1:**
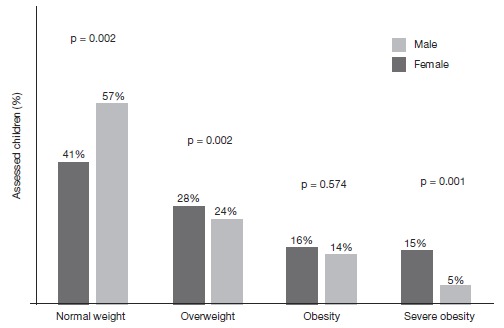
Nutritional status of children aged 6 to 10 years from a private school, according to gender (n = 331). Pelotas (RS), 2012.

Regarding the children's nutritional status, the prevalence of overweight children was 26%, followed by 15% of obese and 10% of severely obese children. Thus, it is noteworthy that half of the study population (51%) had some degree of excess weight. Overweight and severe obesity were more common in boys than in girls (28% of overweight *versus* 24%, *p*=0.002, and 15% of severe obesity *versus* 5%, *p*=0.001, in boys and girls, respectively) (Fig. 1). It was observed that 49% of the children had normal weight, and this condition was more common in girls than in boys (57% *versus* 41%, *p*=0.002). None of the children were classified as thin according to the BMI/age assessment in our sample.

**Figure 2:**
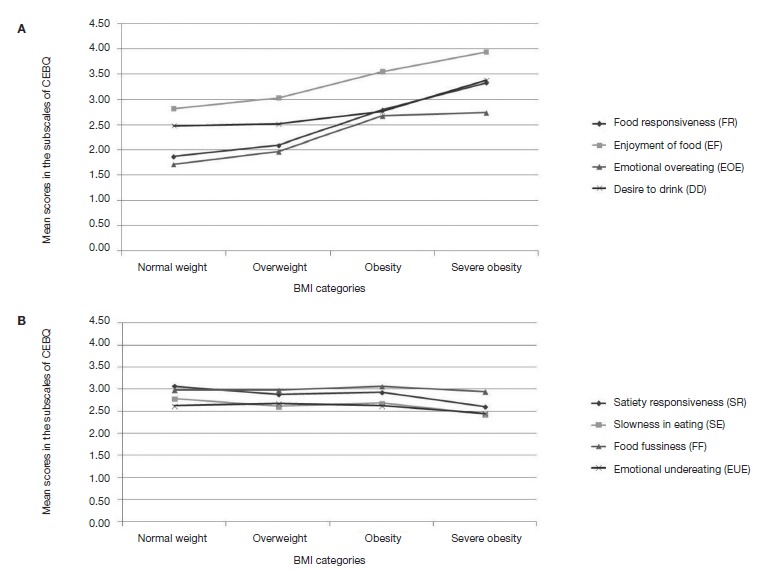
Mean scores for the subscales of "interest in food" (A) and "lack of interest in food" (B) of the CEBQ, according to categories of body mass index of the children (n=331). Pelotas, RS, 2012.

Table 1 shows the scores on the CEBQ subscales according to categories of BMI/age z-score, gender and age group of the children. It was observed that all subscales showed significant association with nutritional status, except for the subscales "Food Fussiness" and "Emotional Undereating" (*p*=0.254 and *p*=0.637, respectively). It was observed that all subscales of "interest in food" had higher scores in the obesity and severe obesity categories (Fig. 2). Conversely, among the subscales that reflected "lack of interest in food", two of them - "Satiety Responsiveness" and "Slowness in Eating" - had the highest scores in the category of normal weight children, whereas for the other two - "Food Fussiness" and "Emotional Undereating" - no significant trend was observed in the variation of scores according to the BMI z-score/age categories (Fig. 2).

In general, the eating behavior was very similar in boys and girls. The only subscale that was different between the genders was the "Desire to Drink", with the mean score being significantly higher in boys than in girls (2.80±1.11 *versus* 2.47±1.07, respectively,* p*=0.005) (Table 1). Eating behavior was very similar in all age groups, and only the subscale "Slowness in Eating" showed a significant difference between age groups, with a decrease in the score of this subscale with increasing age (Table 1).

**Table 1:** Mean ± standard deviation of the CEBQa subscales according to categories of body mass index for age (BMI/age),b gender and age of children.

	Food Responsiveness (FR)	Enjoyment of Food (EF)	Emotional Overeating (EOE)	Desire to Drink (DD)	Satiety Responsiveness (SR)	Slowness in Eating (SE)	Food Fussiness (FF)	Emotional Undereating (EUE)
BMI/age (n=331)								
Normal weight (n=163)	1.87 (0.56)	2.82 (0.78)	1.72 (0.66)	2.48 (1.03)	3.07 (0.60)	2.79 (0.57)	2.98 (0.33)	2.63 (0.92)
Overweight (n=86)	2.10 (0.68)	3.03 (0.75)	1.97 (0.81)	2.52 (1.05)	2.88 (0.52)	2.62 (0.59)	2.98 (0.25)	2.68 (0.82)
Obesity (n=50)	2.80 (0.93)	3.55 (0.81)	2.68 (0.96)	2.76 (1.17)	2.93 (0.42)	2.69 (0.45)	3.06 (0.25)	2.63 (0.82)
Severe obesity (n=32)	3.33 (0.93)	3.94 (0.77)	2.74 (1.02)	3.38 (1.17)	2.61 (0.49)	2.43 (0.46)	2.94 (0.29)	2.45 (0.82)
*p *(ANOVA)^c^	<0.001	<0.001	<0.001	<0.001	<0.001	0.003	0.254	0.637
*p *(Trend test)^d^	<0.001	<0.001	<0.001	<0.001	<0.001	<0.001	0.752	0.450
Gender (n=335)								
Male (n=163)	2.26 (0.83)	3.10 (0.86)	2.12 (0.87)	2.80 (1.11)	2.97 (0.55)	2.72 (0.57)	2.99 (0.28)	2.70 (0.85)
Female (n=172)	2.17 (0.87)	3.08 (0.87)	1.95 (0.90)	2.47 (1.07)	2.94 (0.58)	2.68 (0.54)	2.99 (0.31)	2.58 (0.90)
*p *(ANOVA)^c^	0.377	0.857	0.070	0.005	0.643	0.490	0.894	0.199
Age (n=335)								
<7 years (n=134)	2.15 (0.79)	3.04 (0.90)	1.94 (0.84)	2.56 (1.10)	3.00 (0.57)	2.81 (0.57)	2.97 (0.32)	2.68 (0.86)
7–7.9 years (n=119)	2.26 (0.87)	3.11 (0.90)	2.11 (0.90)	2.76 (1.08)	2.96 (0.54)	2.66 (0.56)	3.00 (0.27)	2.61 (0.86)
≥8 years (n=82)	2.25 (0.90)	3.15 (0.74)	2.08 (0.92)	2.58 (1.14)	2.88 (0.58)	2.55 (0.49)	3.00 (0.28)	2.60 (0.90)
*p *(ANOVA)^c^	0.529	0.602	0.279	0.319	0.313	0.002	0.717	0.751

^a^ CEBQ (Children's Eating Behaviour): questionnaire to assess infant feeding behavior. ^b^ Classification according to cutoffs recommended by the World Health Organization (2007) for children older than 5 years. ^c^ p value by ANOVA. ^d^ p value by the linear trend test.

A multivariate analysis of the association between each of the subscales and the BMI z-score categories adjusted for the children's gender and age and parents' level of schooling was carried out (Table 2). The results obtained in the crude analysis for each of the subscales did not change after the adjustment, and the association between all CEBQ subscales and the BMI z-score categories was maintained, except for the subscales "Food Fussiness" and "Emotional Undereating", as seen in Table 1.

**Table 2:** Linear regression analysis for BMI z-scores (reference category: normal weight) in CEBQ subscales (n = 331).

Subscales	Crude β Coefficient (SE)			*p* ^a^	Adjusted β Coefficient^b^ (SE)			*p* ^a^
	Overweight	Obesity	Severe obesity		Overweight	Obesity	Severe obesity	
Food Response (FR)	0.22 (0.09)	0.93 (0.11)	1.46 (0.13)	<0.001	0.22 (0.10)	0.93 (0.12)	1.49 (0.14)	<0.001
Enjoyment of food (EF)	0.21 (0.10)	0.73 (0.12)	1.11 (0.15)	<0.001	0.21 (0.10)	0.76 (0.13)	1.16 (0.15)	<0.001
Emotional Overeating (EOE)	0.25 (0.10)	0.96 (0.13)	1.02 (0.15)	<0.001	0.21 (0.11)	0.94 (0.13)	1.00 (0.16)	<0.001
Desire to drink (DD)	0.04 (0.14)	0.28 (0.17)	0.89 (0.21)	<0.001	0.05 (0.14)	0.24 (0.18)	0.75 (0.21)	<0.001
Satiety Response (SR)	—0.18 (0.07)	—0.14 (0.09)	—0.46 (0.11)	<0.001	—0.19 (0.07)	—0.14 (0.09)	—0.48 (0.11)	0.002
Slowness in eating (SE)	—0.17 (0.07)	—0.10 (0.09)	—0.36 (0.11)	0.003	—0.15 (0.07)	—0.09 (0.09)	—0.36 (0.11)	<0.001
Food fussiness (FF)	—0.0009 (0.04)	0.08 (0.05)	—0.04 (0.06)	0.254	—0.004 (0.04)	0.09 (0.05)	—0.05 (0.06)	0.480
Emotional Undereating (EUE)	0.05 (0.12)	—0.005 (0.14)	—0.18 (0.17)	0.637	0.03 (0.12)	—0.01 (0.14)	—0.21 (0.17)	0.811

SE, standard error. ^a^ p value at Wald test. ^b^ Analysis adjusted for gender and age of children and parental level of schooling.

## Discussion

The findings of the present study suggest that eating behavior was strongly associated with the child's nutritional status. Children with excess weight had higher scores at all CEBQ subscales that reflect "interest in food", and lower scores at the subscales that reflect "lack of interest in food", when compared to normal weight children. In general, there were no differences in eating behavior between boys and girls, or depending on age.

The children with excess weight showed greater response to food, pleasure in eating, increased food intake due to the emotional state and a greater desire for beverages, and on the other hand, weaker response to satiety and a pattern of faster food intake when compared to normal weight children. Similar results were previously found in studies^4^^-^ 7 ^,^ 13 that used CEBQ to compare the eating behavior in samples of English, Portuguese and Dutch children and adolescents.

It was observed that children with higher BMI/older age had higher scores at the subscales "Pleasure Eating" and "Response to Food", in agreement with studies that show that children who are overweight have increased interest in food and a more pronounced response capacity to the influence of external food attributes such as taste, color and smell.^4^^,^ 6 ^,^ 7 ^,^ 13 ^,^ 22 These two dimensions of eating behavior are also investigated in the Dutch Eating Behavior Questionnaire for Children (DEBQ-C),^10^ in the subscale "External Eating", which indicated that excess weight children scored higher for "external eating" than those with normal weight (p=0.02), similar to the findings of the present study. 

It was verified that children with higher BMI/older age had higher scores at the subscale "Desire to Drink", which reflects the desire of children to carry with them beverages with low nutritional value and high energy density (soft drinks and sweetened juices). Studies^23^^,^ 24 have demonstrated a positive association between consumption of sugary beverages and BMI, suggesting that a decrease in soda consumption could result in a reduction in the number of overweight children. 

Children with excess weight had higher scores at the subscale "Emotional Overeating" when compared to the ones with normal weight, but for the subscale "Emotional Undereating" no significant difference was found between the groups, similar to the findings of the study by Webber et al*.* 13 Our results add to the discussion about the association between emotional eating and nutritional status. Tanofsky-Kraff et al^12^ developed a specific scale to assess the behavior of emotional eating (Emotional Eating Scale adapted for children and adolescents - EES-C), and when studying a sample of young individuals with and without excess weight they found no association between emotional eating and body weight. On the other hand, studies using CEBQ usually show that emotional overeating is positively associated with BMI, whereas emotional undereating is negatively related to BMI.^5^^,^ 6

The CEBQ subscales that reflect "lack of interest in food" ("Satiety Responsiveness", "Slowness in Eating", "Emotional Undereating" and "Food Fussiness") seem to better characterize the eating behavior of underweight children.^4^^-^ 7 ^,^ 13 In our study, even though there were no underweight children, we could observe a significant difference in the scores of the subscales "Satiety Responsiveness" and "Slowness in Eating" between children with normal weight and children with excess weight, although no difference was found for the subscales "Food Fussiness" and "Emotional Undereating".

Children with excess weight had lower scores at the subscale "Satiety Responsiveness" when compared to the normal weight ones, going for the idea that a decrease in response to satiety makes children less capable of regulating food intake, and thus contributes to excess weight gain.^5^^,^ 6 ^,^ 7 ^,^ 13 ^,^ 16 ^,^ 22 Other studies^25^^-^ 27 have indicated that the strategies used by parents to make their children eat a meal or try new foods can hinder the learning of appetite regulation capacity. In this study, overweight children had lower scores at the subscale "Slowness in Eating", demonstrating a faster eating pattern. An experimental study^14^ evaluated the eating behavior of 80 children aged between 8 and 12 years during a test meal carried out in a laboratory, in the presence of the mothers, and demonstrated that overweight children ate faster and with greater bite size when compared to normal weight children. Another similar experimental study by Berkowitz et al^15^ found that the behavior of fast food intake, characterized by a greater number of bites per minute during the meal, was crucial to excessive weight gain and changes in BMI from 4 to 6 years of age, while factors such as calorie consumption rate and/or warnings received by parents during the meal did not affect weight gain.

In general, boys and girls showed a very similar eating behavior in our study, with a difference in mean points only for the subscale "Desire to Drink", in which boys had higher scores. This is in agreement with the Portuguese study,^7^ which evaluated 249 young individuals aged between 3 and 13 years. This behavior has been pointed out among the probable causes for the increase in childhood obesity.^23^^,^ 24 In this study, boys simultaneously had increased interest in sugary drinks and higher prevalence of overweight and severe obesity. Studies have shown no consensus regarding the variation in eating behavior depending on the child's gender.^4^^-^ 6 In the CEBQ validation study,[4] the authors found no differences in behavior between the genders either, and they state that it is probably during adolescence that major differences between boys and girls can be observed, as it is during this phase that girls begin to worry about body self-image, which leads to food restriction attitudes and increased esthetic awareness.^28^^,^ 29

The present study showed no differences regarding the eating behavior according to the children's age range, except for the subscale "Slowness in Eating", in which scores decreased with increasing age, suggesting that older children tend to eat faster. A study^5^ that evaluated young individuals in a broader age range (3-13 years) was able to observe significant differences in all CEBQ subscales according to age, with the exception of the subscales "Emotional Undereating" and "Desire to Drink". Some studies^4^^,^ 5 ^,^ 30 have argued that as children grow up and have the autonomy to choose what they want to eat and how much, their eating behavior tends to undergo changes, among them an increase in the velocity of ingestion.

The relevance of the present study is due to the fact that it was one of the first studies in our country to investigate the psychobiological aspects of child eating behavior through an internationally validated questionnaire (CEBQ). Different from other studies that sent the questionnaires to be filled out by the parents, our study achieved a high response rate (93%). However, this study has some limitations that should be considered. The main limitation concerns the convenience sample used, which was restricted to children attending a single private school in the municipality of Pelotas. Underweight children and lower income groups are not represented in this study, and thus caution is required when generalizing the data found here to other populations. It is worth mentioning that the children's behavior was assessed by the parents' subjective perception, which was used as a "proxy" of the eating behavior, through a questionnaire validated in a sample of Portuguese children, not Brazilian ones. Furthermore, the cross-sectional design carries a limitation regarding the causal inference, as it is not possible to verify whether the assessed eating behaviors were determinants or consequence of excess weight. 

Studies currently investigating factors associated with childhood excess weight still focus on the biological determinants and lifestyle, without considering aspects of eating behavior that may be involved in this process. Our results indicate the existence of several behavioral differences between children with and without overweight, although no differences were found in eating behavior between genders or depending on the age. It is worth mentioning that longitudinal studies are necessary to strengthen the evidence base on the role of eating behaviors in the etiology of obesity and to understand the possible behavioral differences according to the child's gender and age range. The study findings may help the development of effective nutritional interventions to promote healthy eating behaviors among children and reduce childhood excess weight in our country.
