# Supplementary material for: Children's eating behavior: comparison between normal and overweight children from a school in Pelotas, Rio Grande do Sul, Brazil
Source: Rev Paul Pediatr. 2015 Mar;33(1):42–9. doi: 10.1016/j.rpped.2014.11.007 (PMC4436955; doi:10.1016/j.rpped.2014.11.007)
Supplement: Translation

# Translation: Comportamento alimentar infantil: comparação entre crianças sem e com excesso de peso em uma escola do município de Pelotas, RS

**Authors:** Darlise Rodrigues dos Passos, Denise Petrucci Gigante, Francine Villela Maciel, Alicia Matijasevich

**Affiliations:** a Universidade Federal de Pelotas (UFPel), Pelotas, RS, Brasil; b Universidade de São Paulo (USP), São Paulo, SP, Brasil

**Keywords:** Obesidade, Avaliação nutricional, Comportamento alimentar, Criança

## Abstract

**OBJETIVO:**

Avaliar diferenças no comportamento alimentar infantil em função do estado nutricional, do sexo e da idade.

**MÉTODOS:**

O estudo incluiu crianças na faixa de seis a dez anos, de ambos os sexos, de uma escola privada em Pelotas (RS), em 2012. Para avaliar o comportamento alimentar usaram-se as subescalas do questionário Children's Eating Behaviour Questionnaire (CEBQ): resposta à comida (FR), prazer de comer (EF), desejo de beber (DD), sobreingestão emocional (EOE), subingestão emocional (EUE), resposta à saciedade (SR), seletividade (FF) e ingestão lenta (SE). Avaliou-se o estado nutricional por meio do escore-z do IMC/idade.

**RESULTADOS:**

Participaram 335 crianças de 87,9±10,4 meses. Apresentaram eutrofia 49,3% (n=163), sobrepeso 26% (n=86), obesidade 15% (n=50) e obesidade grave 9,7% (n=32). Crianças com excesso de peso tiveram maior pontuação nas subescalas de "interesse pela comida" (FR, EF, DD, EOE, *p*<0,001) e menor pontuação nas subescalas de "desinteresse pela comida" (SR e SE, *p*<0,001 e *p*=0,003, respectivamente), se comparadas com as crianças com peso adequado. Não foram observadas diferenças no comportamento alimentar segundo sexo e idade.

**CONCLUSÕES:**

Observou-se que comportamentos alimentares que refletem "interesse pela comida" estão associados positivamente ao excesso de peso, mas não foi encontrada associação com o sexo e a idade da criança.

## Introdução

O estudo do comportamento alimentar ocupa papel central na prevenção e no tratamento das doenças crônicas associadas a uma alimentação inadequada.^1^ Dentre elas destaca-se a obesidade, que é o principal agravo nutricional enfrentado em países desenvolvidos e em desenvolvimento.^2^ Em 2008, aproximadamente 33% das crianças brasileiras entre cinco e nove anos de idade apresentavam excesso de peso e 14% já estavam obesas.^3^

Estudos têm apontado diferenças em várias dimensões do comportamento alimentar entre crianças com e sem excesso de peso.^4^^,^ 5 ^,^ 6 ^,^ 7 ^,^ 8 ^,^ 9 ^,^ 10 ^,^ 11 ^,^ 12 ^,^ 13 ^,^ 14 ^,^ 15 ^,^ 16 ^and^ 17 Acredita-se que crianças com excesso de peso sejam mais responsivas a estímulos externos presentes no ambiente (por exemplo, sabor e cor dos alimentos), demonstrem maior prazer ao comer e apresentem menor capacidade de resposta à saciedade se comparadas com as crianças com peso saudável, o que faz com que elas comam em maior quantidade e na ausência de fome e demonstrem, assim, um maior interesse pela comida.^4^^,^ 5 ^,^ 11 ^,^ 13 ^and^ 16 Além disso, têm o hábito de comer para lidar com diferentes estados emocionais (alegria, ansiedade e estresse),^4^^,^ 5 ^and^ 13 ingerem frequentemente bebidas açucaradas ao longo do dia e comem mais rapidamente.^14^ ^and^ 15 Por outro lado, crianças com baixo peso parecem ser mais seletivas em relação à alimentação, consomem refeições pequenas, com um número restrito de alimentos e mais lentamente e refletem, assim, um desinteresse pela comida.^4^ ^and^ 13

Sabe-se que o comportamento alimentar começa a ser formado desde os primeiros anos de vida^18^ e os hábitos alimentares da idade adulta estão relacionados com os aprendidos na infância.^19^ Além disso, mudanças de comportamento com o avançar da idade tendem a ser mais difíceis de ser alcançadas.^20^ Essas condições demonstram a importância de se investigar comportamentos alimentares em idades precoces e sugerem que ações voltadas para a promoção de hábitos alimentares saudáveis deveriam incidir com maior ênfase na infância. Face ao exposto, o objetivo do presente estudo foi avaliar diferenças no comportamento alimentar de acordo com o estado nutricional, o sexo e a idade de crianças de seis a dez anos de uma escola privada no município de Pelotas.

## Método

Estudo transversal, feito em uma escola privada do município de Pelotas (RS), de maio a junho de 2012. Foram incluídas no estudo todas as crianças na faixa etária entre seis e dez anos, matriculadas no 1°, 2° ou 3° ano do ensino fundamental, cujos pais ou responsáveis deram autorização para participar da pesquisa (359 crianças). Foram excluídas as crianças sem informação antropométrica.

O desfecho estudado foi o comportamento alimentar, avaliado por meio da percepção subjetiva dos pais acerca do comportamento de seus filhos ao responderem o questionário CEBQ (Children's Eating Behaviour Questionnaire)^4^ traduzido e validado para uma amostra de crianças portuguesas.^5^ Esse questionário contém 35 perguntas divididas em oito subescalas, de modo que quatro subescalas investigam comportamentos que refletem "interesse pela comida" - resposta à comida (FR), prazer de comer (EF), desejo de beber (DD) e sobreingestão emocional (EOE) - e as outras quatro subescalas refletem comportamentos de "desinteresse pela comida" - subingestão emocional (EUE), resposta à saciedade (SR), ingestão lenta (SE) e seletividade (FF). Exemplos de perguntas contidas no questionário são: "Se tivesse oportunidade o meu filho passaria a maior parte do tempo comendo" (FR), "O meu filho adora comer" (EF), "Se tivesse a oportunidade o meu filho passaria o dia bebendo continuamente (refrigerantes ou sucos adoçados)" (DD), "O meu filho come mais quando está ansioso" (EOE), "O meu filho come menos quando está cansado" (EUE), "O meu filho fica cheio antes de terminar a refeição" (SR), "O meu filho come cada vez mais devagar ao longo da refeição" (SE) e "Perante novos alimentos o meu filho começa por recusá-los" (FF). Os questionários foram enviados por meio das professoras para que os pais ou responsáveis pelas crianças os preenchessem e assinalassem as respostas em uma escala de Likert de cinco pontos, conforme a frequência com que seus filhos apresentam cada comportamento. A pontuação varia de 1 a 5: nunca (1), raramente (2), às vezes (3), frequentemente (4) e sempre (5). Soma-se a pontuação das perguntas pertencentes à mesma subescala, de forma que cada subescala apresentará um valor médio e desvio padrão. Nos casos de questões não respondidas, foi feito contato telefônico a fim de obter a informação.

Na escola, foram coletadas as medidas antropométricas de peso e altura, por nutricionistas ou acadêmicos de nutrição previamente treinados e padronizados. As crianças foram pesadas com roupas leves e descalças, em uma balança digital de bioimpedância (Tanita^(r))^ com capacidade de 150 kg e precisão de 100 g. A estatura foi obtida com estadiômetro vertical portátil (Alturexata^(r))^ com 213 cm e precisão de 0,1 cm. O estado nutricional das crianças foi avaliado por meio do escore-z de índice de massa corporal para idade (IMC/I) e classificado, conforme os pontos de corte propostos pela Organização Mundial da Saúde (OMS),^21^ em cinco categorias: magreza, eutrofia, sobrepeso, obesidade e obesidade grave. Para o cálculo do escore-z foi usado o software Anthro Plus^(r)^, da OMS.^21^

A variável idade foi coletada de forma contínua em meses e posteriormente categorizada nas seguintes faixas: < sete anos, entre sete-7,9 anos e oito anos ou mais. Para avaliar a escolaridade dos pais determinou-se o percentual de pais e mães que tinham nível superior completo.

Os dados coletados foram duplamente digitados no EpiData^(r)^ e todas as análises feitas no Stata^(r)^ versão 12.0. Fez-se análise descritiva dos dados por meio de média e desvio padrão para as variáveis contínuas e proporções para as variáveis categóricas. Para comparar a média de pontos obtida em cada uma das subescalas do CEBQ conforme as categorias das diferentes variáveis de exposição (estado nutricional, sexo e faixa etária), usou-se análise de variância (Anova). Foi feito teste de tendência linear para mostrar a variação da pontuação das subescalas do CEBQ nas diferentes categorias do escore-z de IMC. Fez-se a regressão linear para avaliar a associação entre o escore-z de IMC/idade e as médias das subescalas com controle para potenciais fatores de confusão: sexo e idade das crianças e escolaridade dos pais. A variável escore-z de IMC (categorizada) foi relacionada com a variável sexo com tabelas de contingência. Foram calculadas as respectivas prevalências de crianças em cada uma das categorias de escore-z de IMC e comparadas as prevalências entre o sexo feminino e masculino. Os testes estatísticos foram baseados no teste de qui-quadrado. Adotou-se nível de significância de 5%.

O projeto foi aprovado pelo Comitê de Ética em Pesquisa da Faculdade de Medicina da Universidade Federal de Pelotas, sob protocolo n° 26/2012. O termo de consentimento livre e esclarecido foi assinado pelos responsáveis pelas crianças.

## Resultados

Participaram 335 crianças, 93,3% dos alunos matriculados nas séries incluídas no estudo (total de recusas: 6,7%). Verificou-se que 51,3% das crianças eram do sexo feminino, 94,3% tinham cor de pele branca e a maioria tinha pais com nível superior completo de escolaridade (75,7% das mães e 63,7% dos pais, do total de responsáveis que preencheram o questionário). A média de idade da população estudada foi de 87,9 meses (±10,4), os três grupos etários tinham aproximadamente o mesmo tamanho: menores de sete anos (n=134), entre sete-7,9 anos (n=119) e oito anos ou mais (n=82).

Com relação ao estado nutricional das crianças, encontrou-se prevalência de sobrepeso de 26%, seguida de 15% de obesidade e 10% de obesidade grave. Assim, é interessante observar que metade da população avaliada (51%) apresentou algum grau de excesso de peso. O sobrepeso e a obesidade grave foram mais frequentes entre os meninos, quando comparados com as meninas (28% de sobrepeso *versus* 24%, *p*=0,002; e 15% de obesidade grave *versus* 5%, *p*=0,001, entre meninos e meninas, respectivamente) (fig. 1). Detectou-se que 49% das crianças estavam eutróficas, condição mais frequente entre as meninas em relação aos meninos (57% *versus* 41%, *p*=0,002). Nenhuma das crianças avaliadas apresentou magreza pela avaliação do IMC/idade na nossa amostra.

**Figura 1:** Estado nutricional de crianças de seis a dez anos de uma escola privada, conforme sexo (n = 331). Pelotas (RS), 2012.

A tabela 1 apresenta a pontuação obtida nas subescalas do CEBQ segundo as categorias de escore-z de IMC/idade, o sexo e a faixa etária apresentados pelas crianças. Verificou-se que todas as subescalas mostraram associação significativa com o estado nutricional, exceto as subescalas "seletividade alimentar" e "subingestão emocional" (*p*=0,254 e *p*=0,637, respectivamente). Constatou-se que todas as subescalas de "interesse pela comida" apresentaram maior pontuação nas categorias de obesidade e obesidade grave (fig. 2). De maneira oposta, dentre as subescalas que refletem "desinteresse pela comida", duas delas - "resposta à saciedade" e "ingestão lenta" - tiveram maior pontuação na categoria de crianças eutróficas, enquanto para as outras duas -"seletividade alimentar" e "subingestão emocional" - não foi observada tendência significativa na variação dos pontos conforme as categorias de escore-z de IMC/idade (fig. 2).

**Tabela 1:** Média ± desvio padrão das subescalas do CEBQa de acordo com as categorias de índice de massa corporal para idade (IMC/idade)b, o sexo e a idade das crianças

	Resposta à comida (FR)	Prazer de comer (EF)	Sobreingestão emocional (EOE)	Desejo de beber (DD)	Resposta à saciedade (SR)	Ingestão lenta (SE)	Seletividade (FF)	Subingestão emocional (EUE)
*IMC/idade (n* *=* *331)*								
Eutrofia (n = 163)	1,87 (0,56)	2,82 (0,78)	1,72 (0,66)	2,48 (1,03)	3,07 (0,60)	2,79 (0,57)	2,98 (0,33)	2,63 (0,92)
Sobrepeso (n = 86)	2,10 (0,68)	3,03 (0,75)	1,97 (0,81)	2,52 (1,05)	2,88 (0,52)	2,62 (0,59)	2,98 (0,25)	2,68 (0,82)
Obesidade (n = 50)	2,80 (0,93)	3,55 (0,81)	2,68 (0,96)	2,76 (1,17)	2,93 (0,42)	2,69 (0,45)	3,06 (0,25)	2,63 (0,82)
Obesidade grave (n = 32)	3,33 (0,93)	3,94 (0,77)	2,74 (1,02)	3,38 (1,17)	2,61 (0,49)	2,43 (0,46)	2,94 (0,29)	2,45 (0,82)
p^c^	<0,001	<0,001	<0,001	<0,001	<0,001	0,003	0,254	0,637
p^d^	<0,001	<0,001	<0,001	<0,001	<0,001	<0,001	0,752	0,450

*Sexo (n* *=* *335)*								
Masculino (n = 163)	2,26 (0,83)	3,10 (0,86)	2,12 (0,87)	2,80 (1,11)	2,97 (0,55)	2,72 (0,57)	2,99 (0,28)	2,70 (0,85)
Feminino (n = 172)	2,17 (0,87)	3,08 (0,87)	1,95 (0,90)	2,47 (1,07)	2,94 (0,58)	2,68 (0,54)	2,99 (0,31)	2,58 (0,90)
p^c^	0,377	0,857	0,070	0,005	0,643	0,490	0,894	0,199

*Idade (n* *=* *335)*								
<7 anos (n = 134)	2,15 (0,79)	3,04 (0,90)	1,94 (0,84)	2,56 (1,10)	3,00 (0,57)	2,81 (0,57)	2,97 (0,32)	2,68 (0,86)
7-7,9 anos (n = 119)	2,26 (0,87)	3,11 (0,90)	2,11 (0,90)	2,76 (1,08)	2,96 (0,54)	2,66 (0,56)	3,00 (0,27)	2,61 (0,86)
≥8 anos (n = 82)	2,25 (0,90)	3,15 (0,74)	2,08 (0,92)	2,58 (1,14)	2,88 (0,58)	2,55 (0,49)	3,00 (0,28)	2,60 (0,90)
p^c^	0,529	0,602	0,279	0,319	0,313	0,002	0,717	0,751

a CEBQ, Children's Eating Behaviour (questionário para avaliar o comportamento alimentar infantil.

b Classificação conforme pontos de corte preconizados pela Organização Mundial da Saúde (2007) para crianças maiores de cinco anos.

c Valor p pelo teste Anova.

d Valor p pelo teste de tendência linear.

**Figura 2:** Pontuação média das subescalas de "interesse pela comida" (A) e de "desinteresse pela comida" (B) do CEBQ, conforme as categorias do índice de massa corporal das crianças (n = 331). Pelotas, RS, 2012.

De modo geral, o comportamento alimentar foi muito semelhante entre os meninos e as meninas. A única subescala que apresentou diferença entre os sexos foi a "desejo de beber". A média de pontos foi significativamente maior entre os meninos comparados com as meninas (2,80±1,11 *versus* 2,47±1,07, respectivamente, p=0,005) (tabela 1). O comportamento alimentar foi muito parecido em todas as faixas etárias e apenas a subescala "ingestão lenta" apresentou diferença significativa entre as categorias de idade. Houve uma diminuição nos pontos dessa subescala com o aumento da idade (tabela 1).

Fez-se análise multivariada da associação entre cada uma das subescalas e as categorias de escore-z de IMC com ajuste para as variáveis sexo e idade das crianças e escolaridade dos pais (tabela 2). Os resultados obtidos na análise bruta para cada uma das subescalas não se modificou após ajuste e manteve-se a associação entre todas as subescalas do CEBQ e as categorias de escore-z de IMC, exceto para as subescalas "seletividade alimentar" e "subingestão emocional", como havia sido observado na tabela 1.

**Tabela 2:** Análise de regressão linear para os escores-z de IMC (categoria de referência: eutrofia) nas subescalas do CEBQ (n = 331)

Subescalas	Coeficiente ß bruto (EP)			*p* ^a^	Coeficiente ß ajustado^b^ (EP)			*p* ^a^
	Sobrepeso	Obesidade	Obesidade grave		Sobrepeso	Obesidade	Obesidade grave	
Resposta à comida (FR)	0,22 (0,09)	0,93 (0,11)	1,46 (0,13)	<0,001	0,22 (0,10)	0,93 (0,12)	1,49 (0,14)	<0,001
Prazer de comer (EF)	0,21 (0,10)	0,73 (0,12)	1,11 (0,15)	<0,001	0,21 (0,10)	0,76 (0,13)	1,16 (0,15)	<0,001
Sobreingestão emocional (EOE)	0,25 (0,10)	0,96 (0,13)	1,02 (0,15)	<0,001	0,21 (0,11)	0,94 (0,13)	1,00 (0,16)	<0,001
Desejo de beber (DD)	0,04 (0,14)	0,28 (0,17)	0,89 (0,21)	<0,001	0,05 (0,14)	0,24 (0,18)	0,75 (0,21)	<0,001
Resposta à saciedade (SR)	-0,18 (0,07)	-0,14 (0,09)	-0,46 (0,11)	<0,001	-0,19 (0,07)	-0,14 (0,09)	-0,48 (0,11)	0,002
Ingestão lenta (SE)	-0,17 (0,07)	-0,10 (0,09)	-0,36 (0,11)	0,003	-0,15 (0,07)	-0,09 (0,09)	-0,36 (0,11)	<0,001
Seletividade (FF)	-0,0009 (0,04)	0,08 (0,05)	-0,04 (0,06)	0,254	-0,004 (0,04)	0,09 (0,05)	-0,05 (0,06)	0,480
Subingestão emocional (EUE)	0,05 (0,12)	-0,005 (0,14)	-0,18 (0,17)	0,637	0,03 (0,12)	-0,01 (0,14)	-0,21 (0,17)	0,811

EP, erro-padrão.

a Valor p pelo teste de Wald.

b Análise ajustada para o sexo e a idade das crianças e a escolaridade dos pais.

## Discussão

Os achados do presente estudo sugerem que o comportamento alimentar esteve fortemente associado com o estado nutricional da criança. Crianças com excesso de peso apresentaram maior pontuação em todas as subescalas do CEBQ que refletem "interesse pela comida" e menor pontuação nas subescalas que refletem "desinteresse pela comida" quando comparadas com as crianças eutróficas. De maneira geral, não foram observadas diferenças no comportamento alimentar entre meninos e meninas, nem em função da idade.

As crianças com excesso de peso demonstraram ter maior resposta à comida, prazer de comer, aumento da ingestão de alimentos em função do estado emocional e maior desejo por bebidas e, por outro lado, menor capacidade de resposta à saciedade e padrão de ingestão mais rápido quando comparadas com as crianças eutróficas. Resultados semelhantes foram encontrados previamente em estudos^4^^,^ 5 ^,^ 6 ^,^ 7 ^and^ 13 que usaram o CEBQ para comparar o comportamento alimentar em amostras de crianças e adolescentes ingleses, portugueses e holandeses_._

Observou-se que as crianças com maior IMC/idade obtiveram pontuações mais elevadas nas subescalas "prazer de comer" e "resposta à comida", o que coincide com estudos que apontam que crianças com excesso de peso têm interesse aumentado por comida e uma capacidade de resposta mais pronunciada à influência dos atributos externos dos alimentos, tais como sabor, cor e aroma.^4^^,^ 6 ^,^ 7 ^,^ 13 ^and^ 22 Essas duas dimensões do comportamento alimentar também são investigadas no Dutch Eating Behavior Questionnaire for Children (DEBQ-C),^10^ na subescala "comer externo", que indicou que crianças com excesso de peso pontuaram mais para "comer externo" do que aquelas com peso normal (p=0,02), semelhantemente aos achados do presente estudo estudo.

Verificou-se que as crianças com maior IMC/idade obtiveram pontuações mais elevadas na subescala "desejo de beber", a qual reflete o desejo das crianças de carregarem consigo bebidas com baixo valor nutricional e alta densidade energética (refrigerantes e sucos adoçados). Estudos^23^ ^and^ 24 têm demonstrado uma associação positiva entre consumo de bebidas açucaradas e valores de IMC, o que sugere que um declínio no consumo de refrigerantes poderia resultar em uma redução no número de crianças com excesso de peso.

As crianças com excesso de peso exibiram pontuação mais elevada na subescala "sobreingestão emocional" quando comparadas com as eutróficas, mas para a subescala "subingestão emocional" não foi encontrada diferença significativa entre os grupos, semelhantemente aos achados do estudo de Webber et al.^13^ Nossos resultados aumentam a discussão acerca da relação entre ingestão emocional e estado nutricional. Tanofsky-Kraff et al.^12^ desenvolveram uma escala específica para avaliar o comportamento de ingestão emocional (Emotional Eating Scale adapted for children and adolescents - EES-C) e, ao estudar uma amostra de jovens com e sem excesso de peso, verificaram que não houve associação entre ingestão emocional e o peso corporal. Por outro lado, estudos que usaram o CEBQ apontam, de maneira geral, que a sobreingestão emocional está positivamente associada com o IMC, enquanto a subingestão emocional negativamente relacionada com o IMC.^5^ ^and^ 6

As subescalas do CEBQ que refletem "desinteresse pela comida" (resposta à saciedade, ingestão lenta, subingestão emocional e seletividade alimentar) parecem melhor caracterizar o comportamento alimentar de crianças com baixo peso.^4^^,^ 5 ^,^ 6 ^,^ 7 ^and^ 13 Nosso estudo, mesmo não tendo encontrado criança com baixo peso, conseguiu observar diferença significativa na pontuação das subescalas "resposta à saciedade" e "ingestão lenta" entre crianças com excesso de peso e eutróficas, embora para as subescalas "seletividade alimentar" e "subingestão alimentar" nenhuma diferença tenha sido encontrada.

As crianças com excesso de peso tiveram menor pontuação na subescala "resposta à saciedade" quando comparadas com as eutróficas, o que vai de encontro à ideia de que uma diminuição na resposta à saciedade torna as crianças menos capazes de regular o consumo de alimentos e contribui para o ganho de peso excessivo.^5^^,^ 6 ^,^ 7 ^,^ 13 ^,^ 16 ^and^ 22 Estudos^25^^,^ 26 ^and^ 27 apontam que as estratégias que os pais usam para fazer com que os filhos comam a refeição ou experimentem novos alimentos podem dificultar a aprendizagem da capacidade de regulação do apetite. No presente estudo, as crianças com excesso de peso exibiram menor pontuação na subescala "ingestão lenta", o que demonstra um padrão de alimentação mais rápida. Estudo experimental^14^ avaliou o comportamento alimentar de 80 crianças entre oito e 12 anos durante uma refeição-teste em laboratório na presença das mães e demonstrou que crianças com sobrepeso comeram mais rapidamente e com tamanho de mordidas maior quando comparadas com as com peso adequado. Outro estudo experimental semelhante, feito por Berkowitz et al,^15^ verificou que o comportamento de ingestão alimentar rápida, caracterizado por um maior número de mordidas por minuto durante a refeição, foi determinante para o ganho de peso excessivo e nas mudanças no IMC dos quatro para os seis anos, enquanto fatores como taxa de consumo calórico e ou avisos recebidos pelos pais durante a refeição não interferiram no ganho de peso.

De maneira geral, meninos e meninas apresentaram comportamento alimentar muito semelhante em nosso estudo, houve diferença na média de pontos apenas para a subescala "desejo de beber", na qual os meninos tiveram maior pontuação. Isso coincide com estudo português^7^ que avaliou 249 jovens entre três e 13 anos. Esse comportamento vem sendo apontado entre as prováveis causas do aumento da obesidade infantil.^23^ ^and^ 24 No presente estudo, os meninos apresentaram simultaneamente maior interesse por bebidas açucaradas e maior prevalência de sobrepeso e de obesidade grave. As pesquisas ainda não mostram consenso em relação à variação do comportamento alimentar em função do sexo da criança.^4^^,^ 5 ^and^ 6 No estudo de validação do CEBQ,^4^ os autores também não encontraram diferenças de comportamento entre os sexos e discutem que, provavelmente, é durante a adolescência que se observam maiores diferenças entre meninos e meninas, visto ser nessa fase que as meninas começam a se preocupar com a autoimagem corporal, o que leva a atitudes de restrição alimentar e aumento do cuidado com a estética.^28^ ^and^ 29

No presente estudo não foram observadas diferenças no comportamento alimentar conforme a faixa etária das crianças, exceto para a subescala "ingestão lenta", na qual a pontuação diminuiu com o aumento da idade, o que sugere que as crianças mais velhas tendem a comer mais rapidamente. Estudo^5^ que avaliou jovens em uma faixa etária mais ampla (3-13 anos) conseguiu observar diferenças significativas em todas as subescalas do CEBQ conforme a idade, com exceção das subescalas "subingestão emocional" e "desejo de beber". Alguns estudos^4^^,^ 5 ^and^ 30 discutem que, à medida que as crianças crescem e têm autonomia para escolher o que desejam comer e em qual quantidade, seu comportamento alimentar tende a sofrer mudanças, dentre elas um aumento na velocidade de ingestão.

A relevância do presente estudo deve-se ao fato de ser um dos primeiros trabalhos em nosso país a investigar aspectos psicobiológicos do comportamento alimentar infantil por meio de um questionário validado internacionalmente (CEBQ). Diferentemente de outros estudos que usam a metodologia de envio de questionários para preenchimento pelos pais, nosso estudo obteve uma alta taxa de respostas (93%). No entanto, a presente pesquisa tem algumas limitações que devem ser consideradas. A principal delas diz respeito à amostra de conveniência, a qual se restringiu a crianças pertencentes a uma única escola privada do município de Pelotas. Crianças com baixo peso e menor nível de renda não estão representadas neste estudo. Necessita-se, assim, de cautela para a generalização dos dados aqui encontrados para outras populações. Cabe destacar que o comportamento das crianças foi avaliado por meio da percepção subjetiva dos pais, usada como *proxy* do comportamento alimentar, por meio de um questionário validado em uma amostra de crianças portuguesas, e não brasileiras. Além disso, o delineamento transversal acarreta uma limitação na inferência causal, uma vez que não é possível verificar se os comportamentos alimentares analisados foram determinantes ou consequência do excesso de peso.

Pesquisas que investigam fatores associados ao excesso de peso infantil, ainda hoje, centram-se nos determinantes biológicos e do estilo de vida, sem, contudo, considerar aspectos do comportamento alimentar que podem estar envolvidos nesse processo. Nossos resultados apontam a existência de inúmeras diferenças comportamentais entre crianças com e sem excesso de peso, embora não tenha sido encontrada diferença no comportamento alimentar entre os sexos e nem em função da idade. Ressalta-se que estudos com delineamento longitudinal são necessários para fortalecer a base de evidências sobre o papel dos comportamentos alimentares na etiologia da obesidade e para a compreensão das possíveis diferenças comportamentais de acordo com o sexo e a faixa etária da criança. Os resultados aqui encontrados poderão auxiliar no desenvolvimento de intervenções nutricionais efetivas para a promoção de comportamentos alimentares saudáveis entre as crianças e na redução do excesso de peso infantil em nosso país.
